# Canadian Forest Fires and the Effects of Long-Range Transboundary Air Pollution on Hospitalizations among the Elderly

**DOI:** 10.3390/ijgi3020713

**Published:** 2014-05-20

**Authors:** George E. Le, Patrick N. Breysse, Aidan McDermott, Sorina E. Eftim, Alison Geyh, Jesse D. Berman, Frank C. Curriero

**Affiliations:** 1 Department of Health Policy and Management, Johns Hopkins Bloomberg School of Public Health, 615 N. Wolfe Street, Baltimore, MD 21205, USA; 2 Department of Environmental Health Sciences, Johns Hopkins Bloomberg School of Public Health, 615 N. Wolfe Street, Baltimore, MD 21205, USA; 3 Department of Biostatistics, Johns Hopkins Bloomberg School of Public Health, 615 N. Wolfe Street, Baltimore, MD 21205, USA; 4 ICF International, 9300 Lee Highway, Fairfax, VA 22031, USA; 5 Yale School of Forestry & Environmental Studies, Yale University, 195 Prospect Street, New Haven, CT 06511, USA; 6 Department of Epidemiology, Johns Hopkins Bloomberg School of Public Health, 615 N. Wolfe Street, Baltimore, MD 21205-2103, USA

**Keywords:** air pollution, hospitalizations, PM_2.5_, forest fires, global climate change

## Abstract

In July 2002, lightning strikes ignited over 250 fires in Quebec, Canada, destroying over one million hectares of forest. The smoke plume generated from the fires had a major impact on air quality across the east coast of the U.S. Using data from the Medicare National Claims History File and the U.S. Environmental Protection Agency (EPA) National air pollution monitoring network, we evaluated the health impact of smoke exposure on 5.9 million elderly people (ages 65+) in the Medicare population in 81 counties in 11 northeastern and Mid-Atlantic States of the US. We estimated differences in the exposure to ambient PM_2.5_—airborne particulate matter with aerodynamic diameter of ≤2.5 μm—concentrations and hospitalizations for cardiovascular, pulmonary and injury outcomes, before and during the smoke episode. We found that there was an associated 49.6% (95% confidence interval (CI), 29.8, 72.3) and 64.9% (95% CI, 44.3–88.5) increase rate of hospitalization for respiratory and cardiovascular diagnoses, respectively, when the smoke plume was present compared to before the smoke plume had arrived. Our study suggests that rapid increases in PM_2.5_ concentrations resulting from wildfire smoke can impact the health of elderly populations thousands of kilometers removed from the fires.

## 1. Introduction

Forest fires are known to be a major source of air pollutants [1] on a local and a global scale [2–6]. Each year, combustion products from local and distant wildfires impact large populations worldwide [5,7–13]. The atmospheric pollutant that most consistently increases with biomass smoke from wildfires is suspended fine particulate matter (PM), which is commonly associated with increased mortality and morbidity [1,4,14–17]. The PM in biomass smoke consists mainly of black carbon (soot and charcoal particles), organic carbon, sulfates and/or nitrates, potassium carbonate and silica [12,18].

Short-term exposures to fine particulate matter, PM_2.5_ (airborne particulate matter with aerodynamic diameter of ≤2.5 μm) have been associated with increased mortality and morbidity in various communities around the world and in the United States [19–28]. Most studies addressing health impacts of short-term exposures are related to anthropogenically generated PM, which are commonly associated with automobile combustion and industrial practices. There has been a limited but growing body of literature addressing the impact of shorter-term exposure to smoke from forest and bush fires (referred to as wildfires in this paper) [8,9,11,17,29–33]. The majority of these studies have examined the impact on nearby local communities of exposures to wildfire aerosols. Associated health effects include increased emergency department visits and hospital admissions for chronic obstructive pulmonary disease (COPD), bronchitis, asthma and chest pain [7,13,15,34–37]. For example, the San Diego wildfire in October 2003 caused the daily 24 h average PM_2.5_ concentrations to exceed 150 μg/m^3^, and was associated with significant increases in hospital room emergency room visits for asthma, respiratory problems, eye irritation, and smoke inhalation [38], and increased eye and respiratory symptoms, medication use and physician visits in children living in the San Diego area [30]. In Canada, Moore *et al.* estimated that forest fire smoke in 2003 was associated with excess respiratory complaints in Kelowna (Kelowna, BC, Canada) area residents [31].

The wildfire aerosols have lifetimes on the order of many days [12], which allows transport over large distances [4,8,11,39]. While it is clear that local populations are affected by wildfire events, a growing concern is the potential health impact on geographically distant populations, specifically in susceptible groups such as the elderly. Epidemiologic research has identified the elderly, who are more likely to have pre-existing lung and heart diseases, as a population vulnerable to the effects of short-term exposures to air pollution including fine particles [24,40–50].

In July 2002, a dramatic increase in forest fire activity was registered in the province of Quebec, Canada [5]. Specifically, on 7 July 2002 at least 85 fires were burning out of control and destroyed approximately one million hectares of forest that month. The Canadian Forest Services indicated that the major causal factors contributing to these fires were a long period without precipitation and strong winds coming from the north. Lightning and dry conditions sparked fires on 2 July 2002 in two separate regions southeast of James Bay, which is between 200 and 400 miles north of the U.S. border.

**Figure 1.**
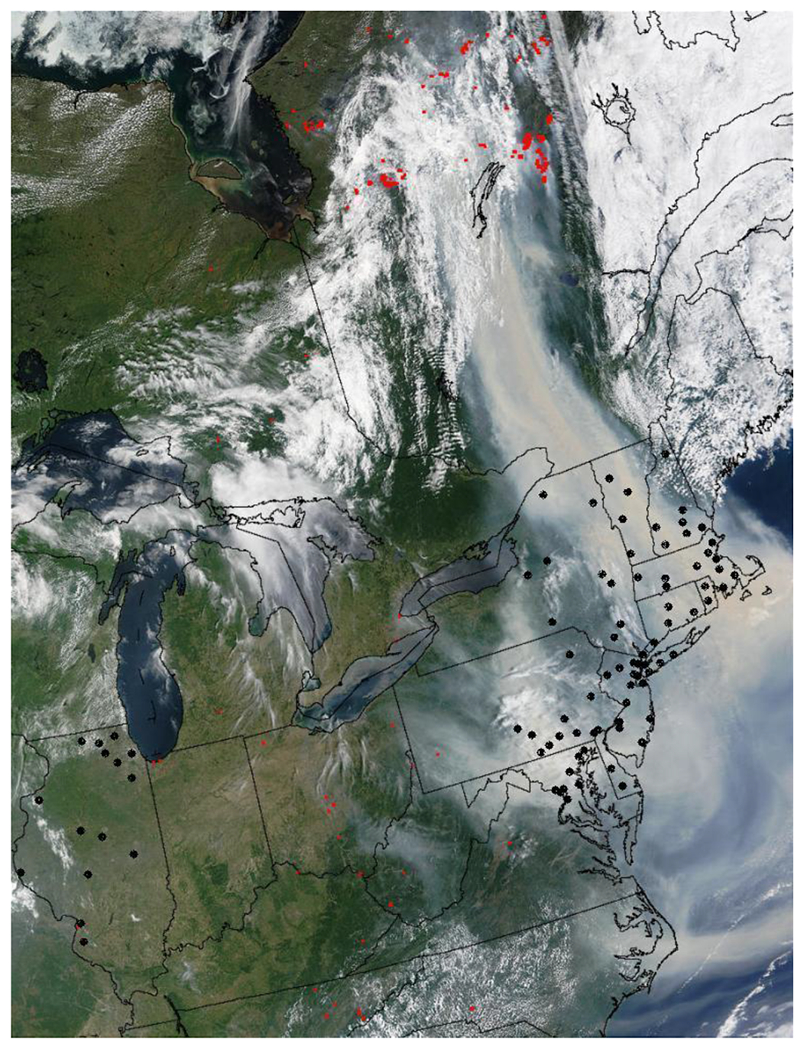
MODIS satellite image taken on 7 July 2002, 10:35 EDT. The red dots mark areas of high forest fire activity. The black dots represent the centroids of counties used in our analysis.

The smoke plume generated from these forest fires had a major impact on air quality across the east coast of the United States during the first week in July 2002 [5,51]. The plume was carried by strong northerly winds from Quebec across the U.S. border covering a distance that extended from north of Montreal to northern Virginia and Maryland [5,51]. Satellite images show the plume on 7 July covering parts of New Hampshire, Vermont, and New York, and diffuse and patchy over the eastern seaboard down to Washington, DC (Figure 1: MODIS satellite image on 7 July 2002 [52]. An air quality study being conducted in Baltimore, MD at that time reported that the 24-hour PM_2.5_ concentrations reached 86 μg/m^3^ on 7 July, resulting in as much as a 30-fold increase in the daily ambient PM_2.5_ concentrations [5] during the peak period of haze over the city. On that day, the highest PM_2.5_ concentration (338 μg/m^3^) was reported in New Hampshire [51]. In many cities in the region (such as New York City and Philadelphia), health advisories were issued for residents with espiratory conditions [53].

This transboundary wildfire smoke episode offered a unique opportunity to evaluate the vulnerability of a susceptible population in the United States exposed to smoke generated from a large-scale wildfires more than a thousand kilometers away. In this paper, we assess the relationship between hospital admissions for individuals 65 years and older and PM_2.5_ concentrations during July 2002 for U.S. states impacted by the Canadian wildfire plume. Unlike previous research that focused on health impacts on populations living near or relatively close to wildfire events, this paper presents one of the first analyses of wildfire smoke health impacts to populations living at great distances, particularly within the U.S., from fires and not previously identified to be at significant risk.

## 2. Methods

### 2.1. Study Area

The study population includes 5.9 million Medicare enrollees aged 65 or older, residing in 81 U.S. counties in the northeastern and mid-Atlantic regions of the U.S. (11 states) and in Illinois. Medicare data included daily health care hospitalization information allowing us to look at changes in hospitalization rates associated with changes in PM air pollution. The study was restricted to 81 plume affected counties that could provide PM_2.5_ data at least once every five days during our study period. These counties are identified in Figure 1. The state of Illinois was chosen as a reference area not affected by the plume for descriptive comparison to the forest fire induced levels of PM_2.5_.

### 2.2. Data Sources

Daily counts of hospital admissions for June–July 2002 were obtained from billing claims for the Medicare National Claims History Files. Each billing claim contains the date of admission, age, sex, place of residence, and cause of hospitalization. Coding for hospitalization is based on the ninth revision of the International Classification of Diseases (ICD-9) [54]. The daily county-specific hospitalization rates were obtained by dividing the daily county-specific admission counts by the daily county-specific number of individuals at risk defined as the number of individuals enrolled in Medicare. The number of individuals at risk, the total Medicare population, varied each day. The daily counts of each health event within each county were obtained by summing the number of hospital admissions for each of the diseases considering both primary and secondary diagnoses. This study was exempt from the Johns Hopkins Bloomberg School of Public Health’s Institutional Review Board because the data for this study did not involve individual identifiers.

The PM_2.5_ air pollution monitoring data were obtained from the U.S Environmental Protection Agency Air Quality System [55]. Temperature and dew point temperature data were gathered from the National Climatic Data Center (NCDC) on the Earth-Info CD database [56]. The analysis was restricted to the 11 northeastern states affected by the plume [53]. Although, carbon monoxide (CO) is a gaseous pollutant commonly associated with forest fires, we did not include it in our analysis because only a quarter of the counties of interest reported CO monitoring data.

### 2.3. Statistical Analysis

County average PM_2.5_ values for use in subsequent regression models were obtained using the geostatistical method known as block kriging. Kriging is a statistical method widely used in the environmental sciences that produces optimal spatial predictions and in the block kriging version produces statistically optimal aggregate level estimates based on point level (monitored) data [57,58]. To implement block kriging, a fine grid is placed over the study area and concentrations predicted at each grid cell using an ordinary kriging approach. Grid points within a specific county are then averaged to provide a single county measure. This allows the more sparsely point monitored PM_2.5_ measurements to be optimally estimated at an aggregate county level by spatially borrowing information from proximal PM_2.5_ measurements. To improve characterization of spatial dependence (a crucial step in the kriging process) and to address issues of edge effects in the block kriging procedure, air monitor values from all inclusive states in the affected region (Figure 1 and including Maine, Virginia, and West Virginia) were utilized. A similar approach was taken for block kriging county estimates in the chosen unaffected reference area of Illinois. Kriging was performed with the “gstat” package in the R Statistical Software [59]. The NCDC weather monitoring network is adequately dense so that county level temperature and dew point temperature estimates were obtained from averaging measurements observed at county specific monitoring stations.

In our primary analysis, we considered specific cardiovascular and respiratory outcomes that have been associated in the literature with short-term exposure to PM_2.5_. This selection would also allow for comparability with previous studies. Outcomes considered were grouped in three broad categories: cardiovascular outcomes (ICD-9 codes (390–459)), respiratory outcomes (460–519) and injuries (800–849) (selected as our control outcome).

In our secondary analysis we targeted specific cardiovascular and respiratory outcomes, that represent endpoints more often associated with acute impacts of short-term exposures to PM_2.5_ in the general population [26,60]: hypertensive disease (401–405), myocardial infarction (410), ischemic heart disease (410–414), acute pulmonary heart disease (415), acute heart disease (410–425), heart rhythm disturbances (426–427), heart failure (428), stroke (430–438), peripheral vascular disease (440–448), and asthma (493), chronic obstructive pulmonary disease (COPD) (490–492, 496), respiratory tract infections (RTI) (464–466, 480–487) and acute respiratory tract infections (460–466) (Table 1).

Satellite imagery and back-trajectory modeling showed that the smoke plume covering the northeastern states on 6–8 July 2002, a Saturday, Sunday, and Monday [5] originated from Province of Quebec. Thus we defined 6–8 July 2002 as our haze period, when air pollution was also at its highest and compared it to a control non-haze period, corresponding to the same days of the preceding week (29, 30 June and 1 July 2002). We chose not to include the same days in the following week as part of the control because of potential persisting effects in the post-haze period [19] The same days of the week were used in this fashion to minimize potential time-varying confounding effects, such as might be expected for weekday *versus* weekend. Within our period of interest, we observed a total of 5772 for all respiratory hospital admissions, and 18,316 hospital admissions for all cardiovascular hospital admissions.

To assess the impact of exposure to PM_2.5_ during the haze period on hospitalization rates for the outcomes of interest, we linked billing claims from Medicare with daily concentrations of PM_2.5_ by county of residence for the Medicare enrollees [60,61] and employed Poisson regression. Regression inference was based on a generalized estimating equation approach (GEE) [62] to account for the possibility of residual temporal autocorrelation in the county-specific hospitalization rates. We assumed an independent working correlation structure for the county-specific daily hospitalization rates, which assumes that the correlation between daily rates decreases with time.

We let $Y_{t}^{c}$ and $\mu_{t}^{c}$ be the observed and expected daily number of cause-specific hospitalizations, $N_{t}^{c}$ be the number of people at risk on day t in county *c*, $X_{t}^{c}$ be the daily average PM_2.5_ for county *c*, $T_{t}^{c}$ and $D_{t}^{c}$ the daily average temperature and dew point temperature for county c. We fit the following log-linear regression model:

(1) $$\text{log}(\mu_{t}^{c})=\beta_{0}+\beta_{1}X_{t}^{c}+\beta_{2}P_{t}^{c}+\beta_{3}T_{t}^{c}+\beta_{4}D_{t}^{c}+\text{log}(N_{t}^{c})$$

with $P_{t}^{c}$ an indicator variable representing haze *versus* non-haze period and $\text{log}(N_{t}^{c})$ representing the regression offset. The parameter *β*_1_ denotes the log relative risk of cause-specific hospitalization associated with one unit (μg/m^3^) increase in daily average PM_2.5_ (block kriged county estimates) adjusted for temperature and dew-point temperature. The parameter *β*_2_ denotes the increase in log relative rate of hospitalizations during the haze period compared to that during the non-haze period.

To account for potential delays in disease incidence after exposure, we also explored single lag models, where we substitute $\beta_{1}X_{t-l}^{c}$ in the county-specific model (1) for $\beta_{1}X_{t}^{c}$, where *l* = 0, 1, 2 days and $X_{t-l}^{c}$ is the PM_2.5_ concentration for county *c* on day *t* at a lag of *l* days [63]. A lag of 0 days corresponds to the association between PM_2.5_ concentrations on a given day and the risk of hospitalization on the same day. We also applied distributed lag models [64,65] to estimate the relative risk of hospitalization associated with cumulative exposure over the current day and the previous two days. The distributed lag models are obtained by substituting $\sum_{i-0}^{2}\beta_{1}X_{t-l}^{c}$ in the county-specific model (Equation (1)) for $\beta_{1}X_{t}^{c}$. Models with a PM_2.5_ by haze period interaction $\beta_{1}X_{t}^{c}P_{t}^{c}$ were also considered allowing for the relative risk of hospitalizations associated with PM_2.5_ exposure to change during the haze period compared to the non-haze period.

## 3. Results

**Figure 2.**
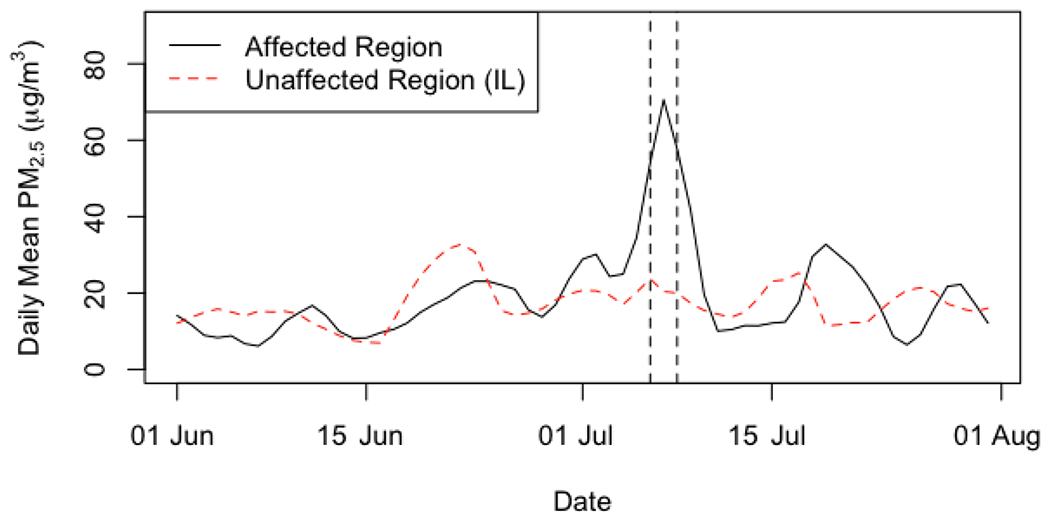
Countywide daily mean PM_2.5_ (μg/m^3^) in the affected and unaffected regions from 1 June to 31 Jul 2002.

Smoke from wildfires in the Quebec region of Canada drifted over the Northeastern and Mid-Atlantic region of the United States on 6–8 July 2002 blanketing much of the area (Figure 1). Figure 2 shows the region wide average daily PM_2.5_ concentrations for the two months surrounding this event from both the affected area and unaffected reference area of Illinois, which are shown to have similar ambient PM_2.5_ concentrations. However, during the identified haze period we observe a substantial spike in PM_2.5_ only in the affected region. The daily averages shown Figure 2 were obtained using 10% trimmed mean to average across monitors after correcting for yearly averages for each monitor; an accepted approach for summarizing longer times series and larger geographic areas of air pollution data [55]. Missing concentrations were imputed using a natural spline interpolation method that accounts for the daily seasonality in PM_2.5_.

**Figure 3.**
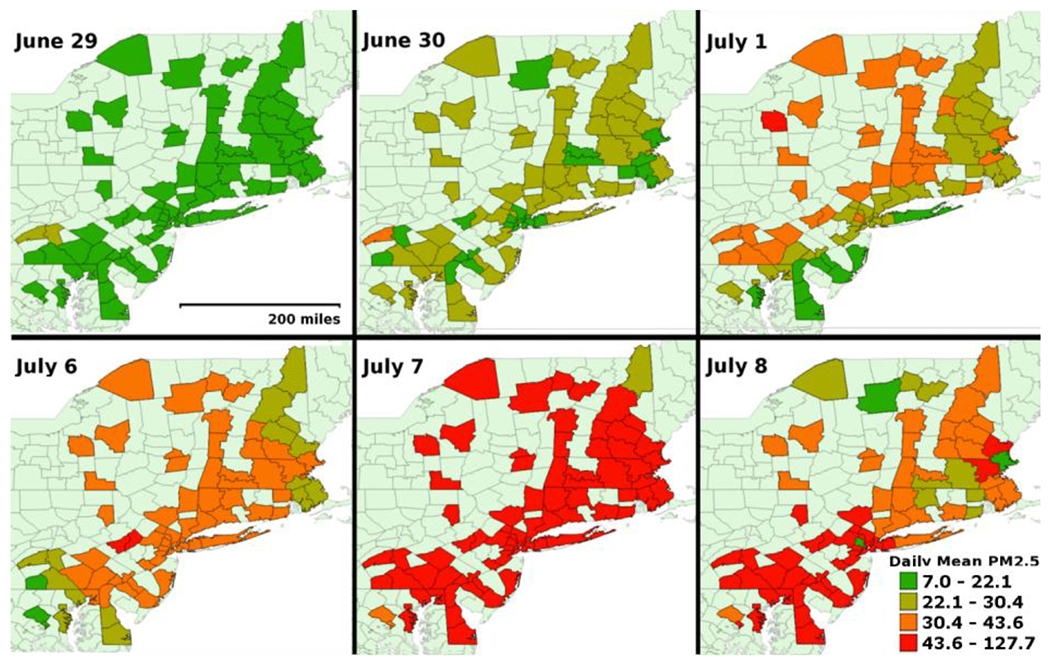
County level block kriged estimated mean PM_2.5_ (μg/m^3^) for the 81 affected counties in non-haze and haze periods. Top Row: Control period between 29 June–1 July designated as non-haze period. Bottom Row: Haze period between 6–8 July.

Figure 3 displays for the affected region the spatial distribution in county specific average PM_2.5_ concentrations (estimated via block kriging) as being substantially higher for days in the haze period compared to the same days in the preceding non-haze period. Table 2 presents summary statistics for these estimated county specific PM_2.5_ concentrations in the affected states and the chosen Illinois reference area during the haze and non-haze periods. The average countywide PM_2.5_ concentrations for 6–8 July 2002 were significantly higher (*p*-value < 0.001) in the haze period (mean 53.0 μg/m^3^, standard deviation SD = 25.0), compared to non-haze period (mean 21.5 μg/m^3^, SD = 10.3 for the affected states. No significant difference in average countywide PM_2.5_ was found in the unaffected reference area of Illinois between the same haze and non-haze periods.

Regression results for the parameter of interest, *β*_2_ from model (1) representing the increase in log relative rate of hospitalization comparing the haze to non-haze period, are presented in Table 3. Results listed in Table 3 are for (exp(*β*_2_) – 1) × 100% representing the percent change in admissions for the three primary outcome categories, all respiratory, all cardiovascular, and the selected control outcome injury. Compared to the non-haze period, this Medicare population had a 49.55% (95% confidence interval (CI): 29.82–72.29) significantly increased rate for respiratory related hospitalizations and a 64.93% (CI: 44.30–88.51) significantly increased rate for cardiovascular related hospitalizations during the haze period compared to the non-haze period, adjusting for weather and PM_2.5_ on the same day (lag 0 model). For the chosen control outcome there was no significant increase in the rate of injury related hospitalizations between the haze and non-haze periods. Single and distributed lag model results show similar significant increases in respiratory and cardiovascular related hospitalizations although not as high as the lag 0 models.

The effect of the forest fire seemed to have a slightly greater impact on cardiovascular than respiratory admissions, a finding contrary to other studies [20,21], which found a higher impact on respiratory admissions than cardiovascular admissions. This may be because of over classification with the use of both primary and secondary discharge codes. However, an increase in cardiovascular and respiratory hospitalizations is consistent with the literature [8,16,24,50,66], though some literature has shown no increase in cardiovascular hospitalizations [2–21] or mortality [67].

**Figure 4.**
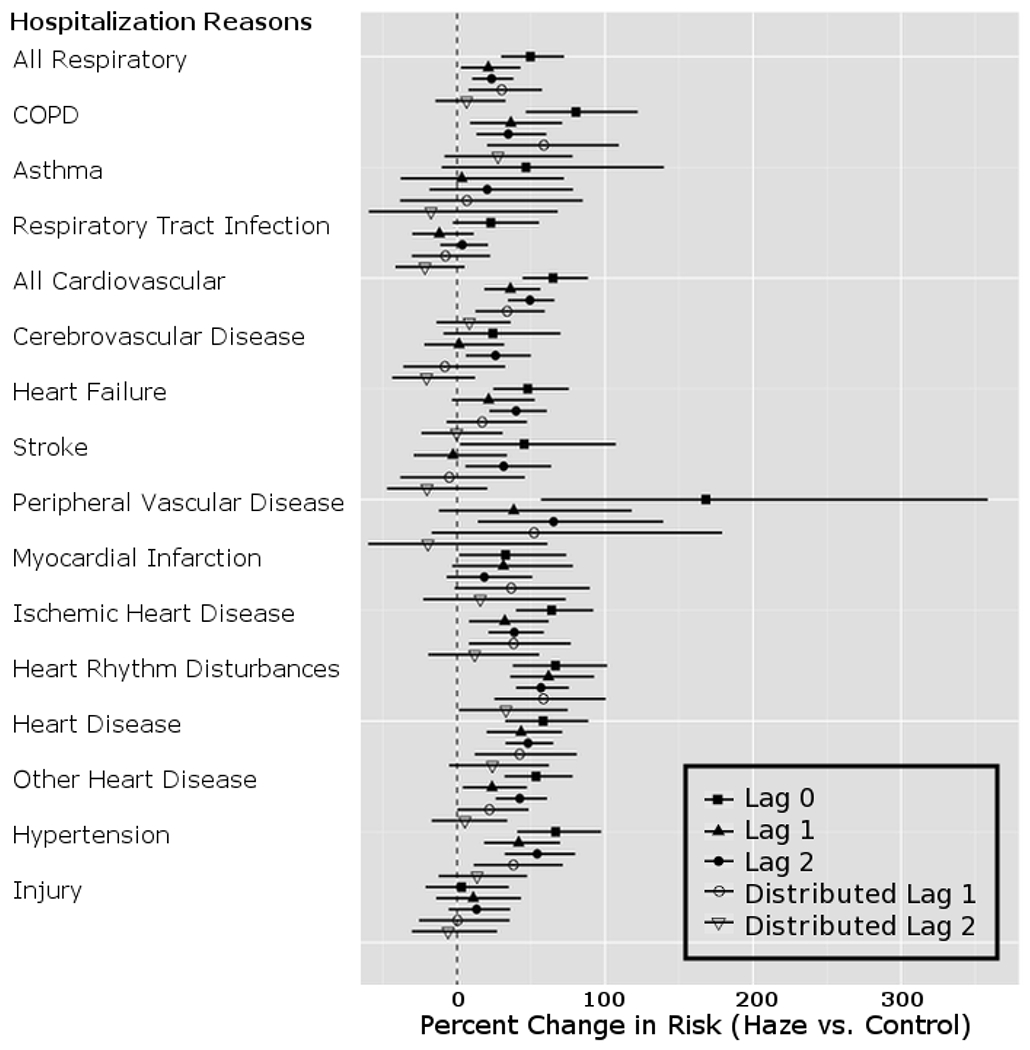
Percent change and 95% CI in hospital related admissions for the haze period compared to the non-haze period in the affected region controlling for PM_2.5_, temperature, and dew point. Note: Percent increase in admissions for the haze period compared to the non-haze period.

Figure 4 displays the percent increase in hospital admissions for the haze period compared to the non-haze period in the affected region for all specific diagnoses of interest with single and distributed lag models. Percent increases in hospitalizations were significantly higher for same day models in all diagnosis groups except for respiratory tract infections, cerebrovascular disease, stroke, and myocardial infarction. For one-day lag models, COPD, heart rhythm disturbances, other heart disease, and hypertension were all significant. The largest change in hospitalization was observed on same day lag models; the magnitude of the effect decreases with increasing inclusion of lag effects. Some of the diagnostic codes, such as asthma, have a low prevalence in hospitalizations and may be more difficult to detect a change in rates of hospitalizations.

Models with the additional PM_2.5_ by haze period interaction term did not reveal consistent or significant results for this interaction effect across many of the outcomes considered (results not shown) suggesting the PM_2.5_ effect not to be the statistically different during the haze and non-haze periods. Results did however continue to reveal the strong significant increase in hospitalizations for the haze period compared to the non-haze period. The lack of evidence supporting a change in PM_2.5_ associated relative risk of hospitalizations during the haze period compared to the non-haze period (PM_2.5_ by haze period interaction) could be due to a combination of several factors including; PM_2.5_ from wildfire sources not any more toxic than non-wildfire sources, the fact that the haze period happened to include both a weekday and the weekend possibly confounding exposure or that there are other drivers of hospitalizations during the haze period that is not entirely explained by PM_2.5_ [68]. Although other studies have shown that wildfire PM is at least as toxic as urban PM [66,68]. Sensitivity analysis was performed increasing the haze period to five, seven, and nine days surrounding 7 July 2002 as well as lagging the haze period when considering models with a lagged PM_2.5_ exposure. Results differed quantitatively, however the overall qualitative interpretations remained consistent. As such all reported and interpreted regressions results were based on model (1) with the predefined three-day haze period.

## 4. Discussion

With the Medicare National Claims History, National Climatic Data Center’s weather data, and EPA’s National Monitoring Network, we conducted an opportunistic study of the effects of wildfire air pollution from the Province of Quebec on the health of the elderly population stretching between New York and the District of Columbia.

The selection of our outcome categories was informed by several considerations. Acute exposure to PM may exacerbate existing pulmonary disease [69–72]. Because COPD is a substantial risk factor for cardiovascular mortality and morbidity [73–76], air pollution exposure may also contribute to cardiovascular risk through exacerbation of COPD symptoms.

We considered several specific cardiovascular and respiratory outcomes that are impacted by inflammatory processes, such as myocardial infarction, stroke, and asthma. The short term effects of exposure to high levels of air pollution are likely to cause inflammatory responses in the lung and release of cytokines with local and systemic consequences [24,77]. Acute effects of PM exposure have also been shown to increase plasma viscosity [25,78].

The log-linear model we used to estimate associations between day-to-day variations in PM_2.5_ (at various lags) and day-to-day variations in the county-level hospitalization rates is typical of time-series analysis [79]. The advantage of the time-series approach is that confounding by individual-level covariates, such as smoking, is not an issue. However, factors that vary with daily pollution exposure, such as weather and co-pollutants, are likely to be confounders in these studies. Time-series analyses typically include nonlinear terms for weather and season [60,63]. One advantage of examining the effects of abrupt increases in PM_2.5_ concentration over a short period of time is that it is unlikely that our analyses were confounded by any seasonal pattern unaccounted for in the model.

Our study focused on a population of interest, the elderly, using a nationally available database of health claims. In interpreting the findings of our analysis, considerations need to be given to the inherent limitations of the data analyzed. Information in the Medicare database is prone to bias due to inaccuracy of claims coding for specific diagnoses [80–83]. In an attempt to reduce misclassification for outcomes of interest, we used primary and secondary diagnosis codes to identify records for inclusion.

In addition, the ambient air pollution data from administrative databases such as EPA’s National Monitoring Network, which have been created for regulatory purposes, only provide limited spatial and temporal coverage. This is an issue typical of air pollution studies relying on publicly available datasets. Also, during the short period when the plume affected the northeast U.S. (three peak days) the number of hospitalizations recorded was small compared to that observed in larger scale time-series studies. The small numbers of hospitalization counts and the limited exposure data reduces the power of our study.

In common with all studies examining the relationship between exposure to air pollution and health that depend on ambient air quality data, our study finding may be biased because of exposure misclassification. In our study all individuals were assumed to be exposed similarly to the corresponding ambient PM measured at EPA monitoring sites. However, some people may have listened to the health advisories (like the ones in New York and Pennsylvania, USA) and retreated indoors during the event. Although, as shown by Sapkota *et al*., some indoor environments were substantially impacted by the elevated ambient PM_2.5_ due to this event [5], the use air conditioning may also have ameliorated the indoor exposure [84].

Wildfires have rapid and substantial impacts on local air quality that elevate ambient PM concentrations well above the norm. The impact of this increased pollution on the health of local populations has been well documented [9,11,13,17,29,30,85–87]. For example, Duclos *et al.* showed a 40% and 30% increase in number of local emergency room visits for asthma and COPD respectively, during 1987 forest fires in northern California [13]. While composition of wildfire smoke has been shown to influence smoke related health outcomes [16], reliance on PM concentrations is common for regional studies because of the fire plume components it is most consistently elevated during smoke events, as opposed to other attributed components like carbon monoxide or nitric oxide [5,32].

Increases in wildfire activity have been linked to warmer spring and summer temperatures resulting from climate change and long-accumulated stocks of combustible vegetation [88,89]. In addition, climate models predict an increase in rising temperatures and regional drying will result in an increase in wildfire activity. Regions like the western U.S. can expect to see considerable increase in wildfire activity [90]. These wildfires may have significant impacts on future air quality and on the health of populations susceptible to the effects of air pollution even those living at a great distance. Our approach is replicable on a larger scale for estimating the health effects of large pollution events resulting from biomass burning over large distances.

## 5. Conclusions

This research adds to the growing body of literature demonstrating the significant impact of transboundary air pollution on public health. Short-term increases in PM_2.5_ concentrations due to the Canadian forest fires in the Province of Quebec show a consistent significant increase in respiratory and cardiovascular hospital admissions for the elderly across the east coast of the U.S. as far south as Washington D.C. These results highlight the public health implications of long-range transport of wildfire-related air pollutants, and raise awareness of the health costs that may be associated with exposures to pollutants generated at remote locations. Preparedness planning by public health and clinical communities is needed to ensure a rapid and adequate response to the challenges that a large wildfire event poses to the health care emergency system. This is especially important as these events are expected to increase as a consequence of global climate change. Knowledge of the potential health risks of wildfire smoke can be translated into local action and used in risk prevention decisions and risk communication such as: educating vulnerable groups of taking their prescribed medication, preparing emergency rooms for an influx of patients with respiratory and cardiac conditions, and advising affected populations on how best to limit their exposures to the smoke.

## Figures and Tables

**Table 1.** Diagnosis Categories.

Reason for Hospital Admission	ICD-9 Codes
Respiratory Outcomes	(460–519)
Chronic Obstructive Pulmonary Disease (COPD)	(490–492, 496)
Asthma	(493)
Respiratory Tract Infections	(464–466, 480–487)
Cardiovascular Outcomes	(390–492, 496)
Cerebrovascular Disease	(430–438)
Heart Failure	(440–448)
Stroke	(410)
Peripheral Vascular Disease	(410–414)
Myocardial Infarction	(426–427)
Ischemic Heart Disease	(426–428)
Heart Rhythm Disturbances	(46–427)
Heart Disease	(426–428)
Other Heart Disease	(420–425, 428)
Hypertension	(401–405)
Injury	(800–849)

**Table 2.** Summary statistics for the block kriged estimated county average PM_2.5_ (μg/m^3^) for the plume affected area and Illinois reference area both stratified by the haze and non-haze periods. Data were pooled over the three days comprising both the haze and non-haze periods.

Summary Statistics	Affected Region		Unaffected Region (IL)	

	Haze	Non-Haze	Haze	Non-Haze
**Min**	17.6	7.0	12.2	15.2
**25th %tile**	35.6	13.7	16.0	19.0
**Median**	43.1	22.6	20.7	20.7
**Mean**	53.0	21.5	22.4	20.8
**75th %tile**	69.1	25.3	29.7	22.1
**Max**	127.7	43.7	33.0	28.4
**SD**	25.0	10.3	7.2	5.0

**Table 3.** Percent change in hospital admissions for the haze period compared to the non-haze period in the affected region controlling for PM_2.5_, temperature, and dew point. Bolded model lag types denote significant change in hospital admissions at the 0.05 level.

Hospitalization Codes	PM_2.5_ Model Lag	Percent Change	95% Confidence Interval
All Respiratory	**Lag 0**	49.55	(29.82, 72.29)
	**Lag 1**	21.14	(2.69, 42.90)
	**Lag 2**	23.36	(10.20, 38.10)
	**Dlag1**	30.20	(7.66, 57.46)
	Dlag2	6.48	(−14.63, 32.81)

All Cardiovascular	**Lag 0**	64.93	(44.30, 88.51)
	**Lag 1**	36.06	(18.43, 56.32)
	**Lag 2**	49.28	(34.39, 65.81)
	**Dlag1**	33.85	(12.45, 59.33)
	Dlag2	8.31	(−13.89, 36.24)

Injury	Lag 0	3.04	(−21.31, 34.92)
	Lag 1	10.97	(−14.04, 43.27)
	Lag 2	13.23	(−5.64, 35.89)
	Dlag1	0.37	(−25.69, 35.55)
	Dlag2	−6.04	(−30.50, 27.02)
